# Adults’ visual recognition of actions simulations by finger gestures (ASFGs) produced by sighted and blind individuals

**DOI:** 10.1371/journal.pone.0214371

**Published:** 2019-03-28

**Authors:** Dannyelle Valente, Amaya Palama, Jennifer Malsert, Guillemette Bolens, Edouard Gentaz

**Affiliations:** 1 Faculty of Psychology and Educational Sciences, University of Geneva, Geneva, Switzerland; 2 Department of Developmental Psychology, University of Lumière Lyon, Bron, France; 3 Faculty of Humanities, University of Geneva, Geneva, Switzerland; Curtin University, AUSTRALIA

## Abstract

The present study examines the visual recognition of action simulations by finger gestures (ASFGs) produced by sighted and blind individuals. In ASFGs, fingers simulate legs to represent actions such as jumping, spinning, climbing, etc. The question is to determine whether the common motor experience of one’s own body is sufficient to produce adequate ASFGs or whether the possibility to see gestures from others are also necessary to do it. Three experiments were carried out to address this question. Experiment 1 examined in 74 sighted adults the recognition of 18 types of ASFGs produced by 20 blindfolded sighted adults. Results showed that rates of correct recognition were globally very high, but varied with the type of ASFG. Experiment 2 studied in 91 other sighted adults the recognition of ASFGs produced by 10 early blind and 7 late blind adults. Results also showed a high level of recognition with a similar order of recognizability by type of ASFG. However, ASFGs produced by early blind individuals were more poorly recognized than those produced by late blind individuals. In order to match data of recognition obtained with the form that gestures are produced by individuals, two independant judges evaluated prototypical and atypical attributes of ASFG produced by blindfolded sighted, early blind and late blind individuals in Experiment 3. Results revealed the occurrence of more atypical attributes in ASFG produced by blind individuals: their ASFGs transpose more body movements from a *character-viewpoint* in less agreement with visual rules. The practical interest of the study relates to the relevance of including ASFGs as a new exploratory procedure in tactile devices which are more apt to convey action concepts to blind users/readers.

## Introduction

Gestures are movements made by the hand or body, meant to express or clarify spoken content [1–3]. They can be codified into an autonomous language, as is the case with sign languages used by deaf communities [4]. Various studies have been conducted to describe and classify the diversity of gestures in sighted communities [1, 3, 5, 6]. In *Why We Gesture*, McNeill [2] posits that we gesture to orchestrate speech. Some gestures are used to emphasize prosody and match the rhythm of speech (*beat gestures*) while others represent attributes of concrete objects, spatial relationships and actions (representational gestures) [5]. Representational gestures are used, for example, to describe the width of a container, the size of a person or to simulate the gait of an animal.

In a perspective of embodied cognition, Hostetter and Alibali [5] support the idea that gestural activity emerges from the activation of visual and motor imagery. The degree to which each type of imagery is involved is related to the point of view used by the speaker when he or she gestures. Two points of view are principally identified, *character-viewpoint* and *observer- viewpoint* [1]. The occurrence of these points of view varies according to the profile of subjects and their capacity to think through imagery. A *character-viewpoint* is produced as though the speaker’s body is the body of the character [5], for example when a speaker mimicking the character’s manual actions (throwing, pounding, etc.). The speaker might also use his/her hand or fingers to show a non-manual action, for example when he/she moves his/her hand up and down to simulate the legs of a cat [1]. *Observer-viewpoint* gestures depict the motion involved in a scene observed at a distance, for example when a speaker simulates with the hand a car that climbs a sloping street. Gestures produced from a *character-viewpoint* result more from motor imagery than *observer-viewpoint* gestures, which principally result from visual imagery.

The present study examined a special category of representational gestures that simulates leg movements using the index and middle fingers. Assessing the production of action simulations by finger gestures (ASFGs) in the absence of sight has a practical interest. We studied the relevance of including ASFGs to illustrate the contents of tactile books for the blind. To explore the illustration, children’s fingers act as the two legs of a character. In our design idea, illustrations use unfolding pop-up mechanisms to produce a miniature 3D scene, upon which the two fingers/legs perform various actions: jumping on a trampoline, climbing stairs, etc. In most cases, tactile books for the blind contain 2D tactile illustrations that are more or less directly transferred from visual illustrations, formed by using raised lines or areas of low relief textures. Several studies have shown that these can be hard for children and adults with visual impairments to understand [7–12]. Linked with embodied views of perception and concepts [13–15] we hypothesized that incorporating body experiences into the book could be a promising way to better adapt illustrative contents for blind readers. Embodied approaches to cognition argue that our sensorimotor experiences with real objects contribute to perceptual and conceptual processes. For example, the concept *stairs*, rather than being an abstract and arbitrary representation of its components, is composed of the simulation of our real experience of climbing and going down stairs [13]. In the same way, studies have shown that reactivation of motor components involved in interactions with objects (enactment effect) can facilitate learning and memorization of concepts [16, 17]. These components can also help in processing contents in the context of blindness. Basing illustrations on sensorimotor rather than visual components also has the advantage of being based on experiences that are possibly the same for sighted and blind children [18, 19].

In order to validate this idea, it is first necessary to know whether a simulation process that transposes the experiences of the legs by imitating their actions with the fingers is meaningful for blind as well for sighted individuals.

Regarding more generally the occurrence of gestures in congenitally blind persons, studies showed that they produced gestures when they spoke, even though they had never seen gestures and had no experience with their communicative function [20–22]. Results suggested that gestures may serve a cognitive function for the speakers themselves, beyond their communicative value. However, the gestural activity of the blind or individuals with visual impairment seems to differ from that of sighted. They use more adaptors (continuous body touching with no manifest relation with their speech) than illustrator or representational gestures linked to the content of the conversation [23–25].

The use of two fingers to simulate the legs of a character, a special category of gestures that will be examined in our study, is part of some games in infancy (nursery rhymes, finger soccer, etc). “Action Simulations by Finger Gestures–ASFG” are also included in the sign language of deaf people to express concepts like climbing stairs, sitting or jumping (Spreadhesign dictionary, European Center of Deaf Language). However, no studies have examined whether individuals spontaneously produce similar patterns of ASFGs. If so, similar prototypical patterns of ASFGs would be observed in blindfolded sighted. Therefore, the comparison between early blind, late blind and blindfolded sighted could allow us to evaluate the role of visual experience in the production of ASFGs. If visual representations and visual experience play a role, similar prototypical patterns of ASFGs would be observed only in late blind and blindfolded sighted. If visual representations and visual experience do not play a role, similar prototypical patterns of ASFGs would be observed in early blind, late blind and in blindfolded sighted.

Because in ASFGs the two fingers are used as a replacement of the legs, we can expect a strong involvement of motor imagery in the process. But individuals also execute gestures with a communicative purpose. Thus, visual experiences could be necessary to convey norms of gestural expressivity learned by face-to-face interactions. These norms can relate to an *Observer-viewpoint* of the gesture [5]. Because early blind individuals cannot, from birth or shortly thereafter, see others’ gestures, they cannot learn these norms. Thus, if ASFG produced by early blind individuals are recognized in the same way as ASFG produced by late blind and sighted individuals, this would provide compelling evidence of a stronger involvement of motor imagery than visual imagery in this type of gesture.

Three experiments were carried out to examine the occurrence of “Action Simulations by Finger Gestures–ASFG” in blind and sighted adults and their visual recognition by adults. The aim of Experiment 1 was to examine, in 74 sighted adults, the visual recognition of 18 types of ASFGs produced by 20 blindfolded sighted adults. Experiment 2 investigated the role of visual experience in the production of the types of ASFGs and their visual recognition. Thus, it examined in 100 other sighted adults visual recognition of the same 18 types of ASFGs presented in Experiment 1, but this time produced by 10 early blind and 7 late blind adults. In Experiment 3, two independent judges analysed prototypical and atypical attributes of ASFG produced by blindfolded sighted, early blind and late blind individuals.

## Experiment 1: Visual recognition of ASFG produced by blindfolded sighted adults

In Experiment 1, ASFGs produced by 20 blindfolded sighted adults were videotaped and presented to 74 sighted judges in an online visual recognition task. Because these gestures are based on the same perceptual experience for sighted individuals who produce these stimuli and the sighted individuals who should recognize them, we hypothesized that ASFGs produced by blindfolded sighted participants will obtain very high rates of recognition. However, some errors of interpretation can be expected among actions which have a similar appearance, such as Squatting and Sitting or Skating and Skiing.

## Method

### Participants

A total of 80 sighted adults (48 women and 32 men, age range 20–70 years) took part in a online visual recognition task consisting of videos of ASFGs produced by blindfolded sighted. Because the task design used an online survey (produced with Quatrics platform) for the final sample, we took into account only those participants who gave a response for more than 90% of the videos. Considering also the hypothesis that subjects can complete the survey without performing the task seriously or attentively, we excluded participants for whom the mean of recognition was less than two standard deviations (2 SD) away from the global mean of recognition. Thus, the final sample was composed of 74 sighted adults participants (44 women and 30 men, age range 20–70 years). All participants are francophone although their nationalities vary (70% french, 22% swiss and 8% other nationalities). French is the native language for 91% of them. Participants’ responses were collected anonymously.

### Stimuli: Production of 18 ASFGs by blindfolded sighted adults

Twenty blindfolded sighted adults (16 women and 4 men; mean age = 27.5 years, SD = 3.48, range = 22–36 years) were asked to produce ASFGs of 18 actions. They were recruited at the University of Geneva. The study was conducted with the written consent of each participant, in accordance with Declaration of Helsinki and controlled and authorized by the Swiss Ethics Committee.

Blindfolded sighted adults were videotaped individually with a Sony HDR–CX 220 camera facing them. They were asked to simulate 18 actions, verbally announced by the experimenter, using the index and middle fingers of their right hand. Between actions, participants were to go back to the resting position (hands placed flat on the table). This procedure limited the influence of the final spatial positioning of an action on the next one. The action presentation order was randomized across subjects. No constraint in terms of space was imposed. Subjects were free to raise and move their hand in all directions and to use the left hand as a support for the gesture if necessary. This study respects ethical principles for research involving human subjects (World Medical Association Declaration of Helsinki) and was approved by the Swiss Ethics Committee on reseach involving humans. The individuals described in this manuscript have given written informed consent (as outlined in the PLOS consent form) to publish these case details.

Table 1 presents actions used in the preparation of stimuli. The list encompasses the broadest range of actions that it is possible to simulate with finger gestures. Conjointly with designers at Les Doigts Qui Rêvent, a French publishing house specialized in the production of tactile books for blind children, we selected ASFGs which would be technically possible to include in a future tactile book.

**Table 1:** Production of stimuli: List of actions.

1	Playing on a swing	7	Playing leapfrog	13	Turning in place
2	Kicking a ball	8	Jumping on one leg	14	Skating
3	Pedaling	9	Jumping off a step	15	Skiing
4	Climbing	10	Squatting	16	Going down stairs
5	Sliding on a toboggan	11	Sitting	17	Climbing stairs
6	Jumping on a trampoline	12	Turning on a merry-go-round	18	Walking backwards

**Fig 1:**
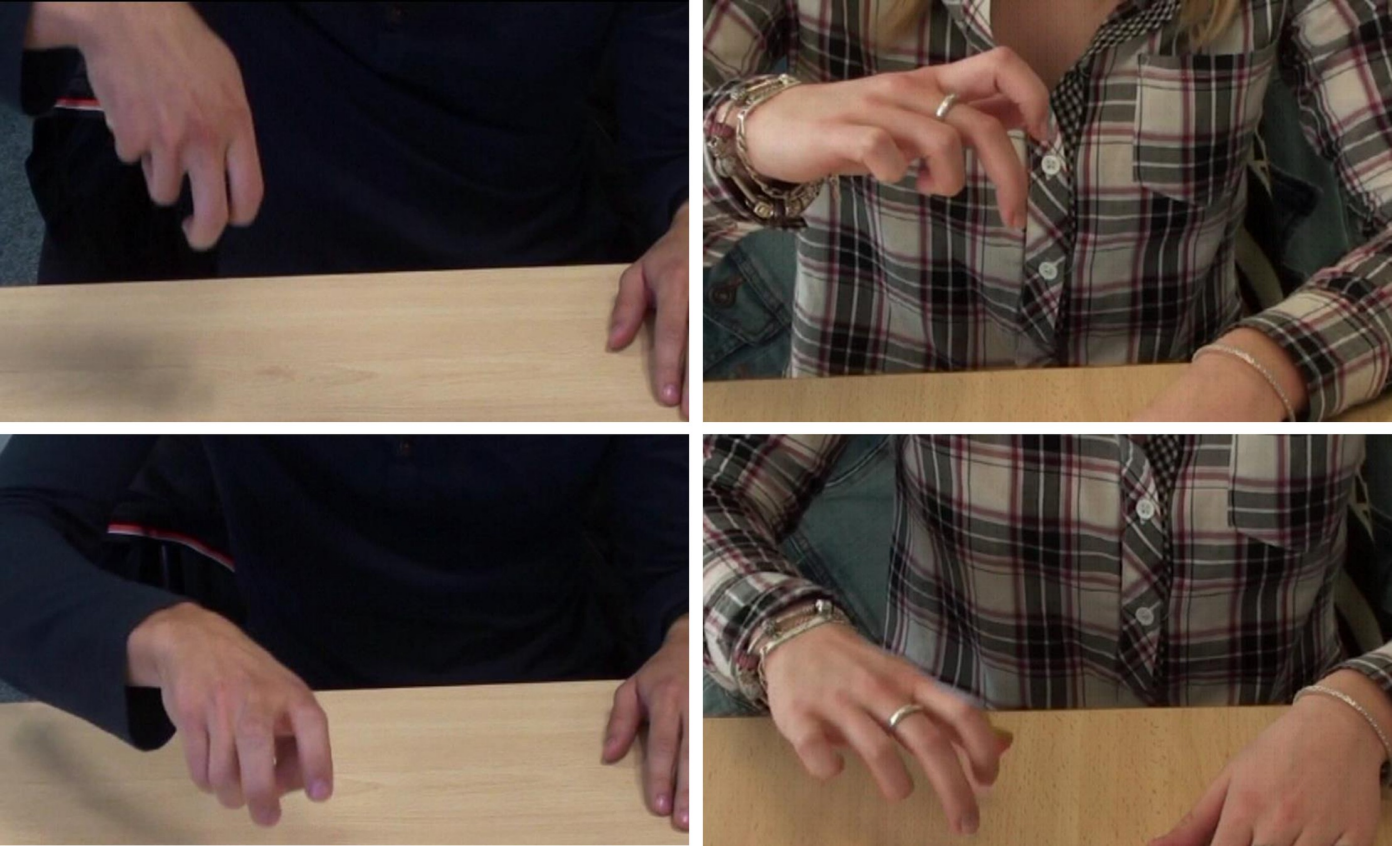
Examples of stimuli. ASFGs of Playing on a swing and Climbing stairs produced by blindfolded sighted participants. Videos of ASFGs are available at: https://www.youtube.com/watch?v=zr2Rr28vzoE and https://www.youtube.com/watch?v=Uzzly9EUZcA.

With CyberLink Power Editor 14, videos of 3 seconds were prepared for each ASFG (Fig 1); each of the 18 actions was produced by 20 encoders (i.e 360 videos at total). All 360 videos were included in the visual recognition task.

### Experimental condition of the visual recognition task

The task of visually recognizing the AFSGs (360 stimuli) was conducted using an online survey organized in four blocks. A total of 20 different sighted participants viewed each block. The online survey procedure was chosen to obtain the most varied profiles of decoders, totally naïve regarding the study’s hypothesis. Content was divided in 4 blocks no longer than 30 minutes each to minimize the risk the survey would be dropped, often important in online survey platforms. Each block contained a total of 90 videos, including sequences of each of the 18 actions in equal number (18 actions x 5 encoders).

We instructed blindfolded sighted participants to watch each video and click the action that they thought the person had simulated with their fingers. The response measure was a multiple-choice method with 18 choices (e.g. 18 actions included in the study). Presentation of videos was randomized across decoders.

### Data analysis of visual recognition task

The percentages of correct recognition as well as the percentages of other meanings attributed to the action were measured. Among the 360 ASFGs, a total of 20 were excluded from subsequent analysis because they had a mean recognition rate less or more than two standard deviations (2 SD) away from the mean recognition rate per action. To maximize approximation to normality for generalized linear model analysis, arcsin transformation was performed on rates of correct recognition [26] (data in S1 Dataset). Results from both statistical analysis (with ratios data and using arcsin transformation) will be presented.

Preliminary main effects ANOVA showed no significant effect of gender (Ratio: *F*(1, 68) = .86, *p* = .35, η_p_^2^ = .01/Arcsin transformation: F(1, 68) = .67, *p =* .41, η_p_^2^ = .01) on rates of recognition of sighted participants (data in S1 Dataset). Consenquently, data were further collapsed across this factor.

In order to control block effect, an 18 (each action) x 4 (survey response blocks) factorial ANOVA was performed on the mean of action recognition with the actions as within-subjects factor and blocks as between-subjects factors. Block conditions were significant, but only using arcsin transformations (Ratio: *F*(3, 268) = 2.30, *p* = .07, η_p_^2^ = .02/Arcsin transformation: *F*(3,268) = 4.23, *p* = .005, η_p_^2^ = .04). However the interaction between actions and blocks conditions was not significant in both cases (Ratio: *F*(51, 268) = .57, *p* = .99, η_p_^2^ = .09/Arcsin transformation: *F*(51, 268) = .66, *p* = .95, η_p_^2^ = .11). Given the fact that the rate of recognition of all actions is the same for all blocks, results were further collapsed across blocks.

A one-way ANOVA was performed on the mean of action recognition (ratios data and after arcsin transformation) with the actions as within-subjects factor. A cross-tabulation was also performed to examine the relationship between the original intended meaning of actions (i.e., ASFG produced) and the meaning attributed to the ASFG by participants (i.e., ASFG chosen from the list of possible answers). The significance threshold was .05; effect sizes are given in partial eta-squared η for ANOVAs.

## Results and discussion

**Fig 2:**
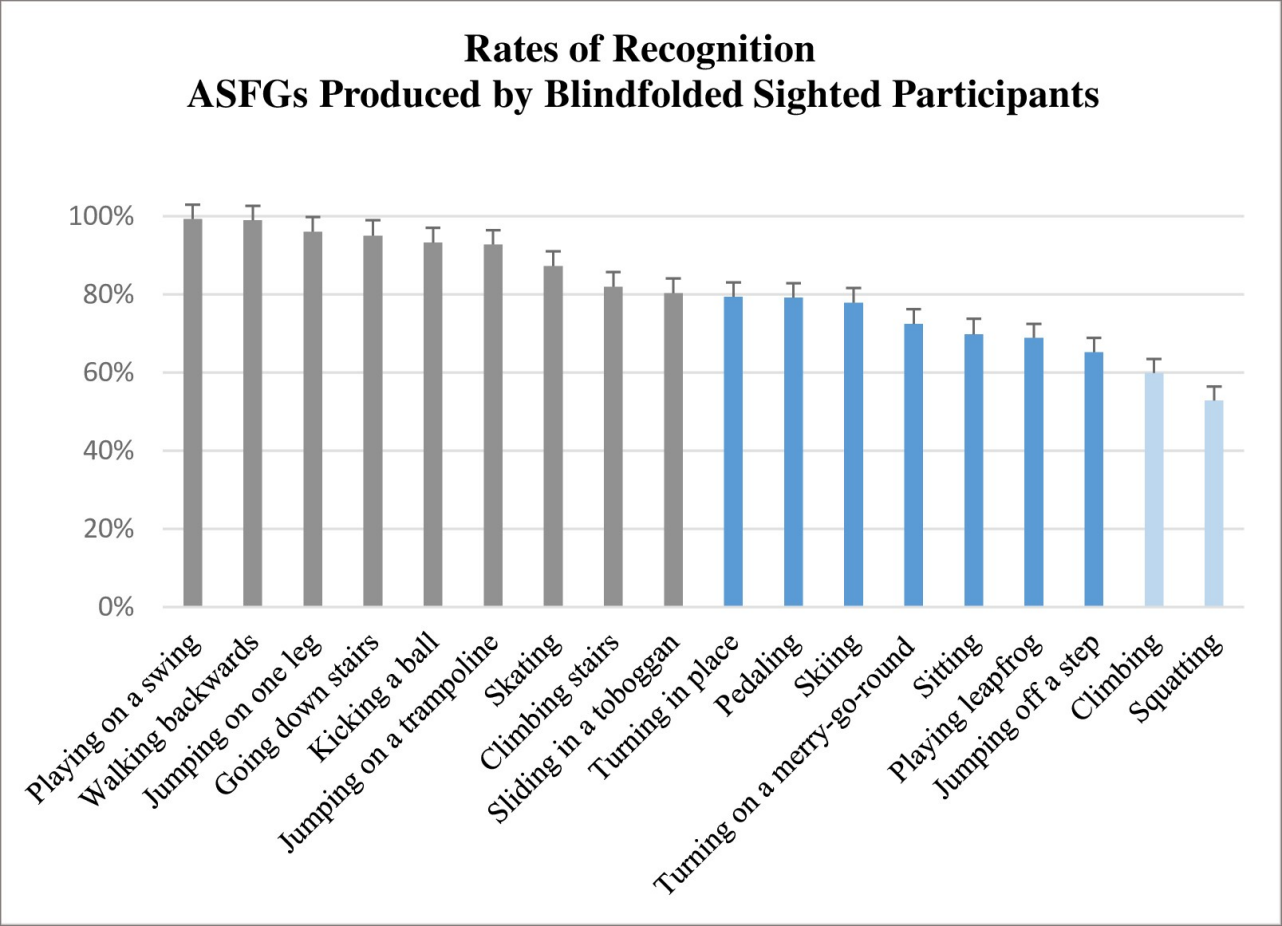
Rates of visual recognition (N = 74) for ASFGs produced by blindfolded sighted individuals (N = 20). Colors differenciate rates between 80–100% (grey), between 60–80% (dark blue) and between 60–40% (light blue). The vertical bars represent positive standard deviations. Results with arcsin transformation is available in S1 Appendix.

To facilitate readability, only means of ratios data will be presented in this section. The overall results with ratios data and arcsin transformation are available in S1 Appendix. On average, action simulations produced by blindfolded sighted were very well recognized (M = 80.5%, SD = 13.8%). An 18 (each action) one-way ANOVA by means of recognition showed a significant effect for action condition (Ratio: *F*(17, 322) = 14.16, *p* < .001, η_p_^2^ = .42/Arcsin transformation: *F*(17, 322) = 20.33, *p* < .001, η_p_^2^ = .51). Fig 2 contains percentages of recognition for each action. We observed that mean rates of recognition were very high for most of these: 9 simulated action simulations were recognized at a rate between 80–100% (grey bars), 7 between 60–80% (dark blue bars) and only 2 between 60–40% (light blue bars).

A cross-tabulation (Table 2) showed relationships between the intended meanings and attributed meanings for the 9 actions recognized at less than 80%. We observed that among the 12 incorrect meanings attributed at a level which was above the level of chance (in grey), the vast majority are in the same category as the intended meaning of that ASFG. For instance, in the category *To turn*, the incorrect meaning *Turning on a merry-go-round* is attributed at a level of 11,4% to *Turning in place*. Inversely, an incorrect meaning *Turning in place* is also attributed at a level of 21,4% to *Turning on a merry-go-round*. Similar results were founded in other actions within the same category, such as *Skating* and *Skiing* or *Playing leapfrog* and *Jumping off a Step*.

**Table 2:** Cross-tabulation of ASFG produced by blindfolded sighted individuals (Columns) and the meaning attributed by participants (Rows).

	Turning in place	Pedaling	Skiing	Turning on a merry-go-round	Sitting	Playing leapfrog	Jumping of a step	Climbing	Squatting
Playing on a swing	0.3%	0.9%	0.6%	0.3%	1.3%	-	-	-	-
Walking backwards	0.3%	-	-	-	0.6%	-	-	0.3%	-
Jumping on one leg	0.9%	2.6%	0.3%	-	-	4.9%	11.7%	-	-
Going down stairs	0.6%	3.2%	-	-	-	0.3%	0.0%	1.6%	-
Kicking a ball	1.1%	0.6%	-	-	-	0.0%	0.3%	-	-
Jumping on a trampoline	-	1.1%	-	-	0.3%	5.2%	3.4%	-	-
Skating	6.3%	2.9%	17.2%	6.5%	-	-	-	0.5%	-
Climbing stairs	-	4.6%	-	-	-	-	-	33.8%	-
Sliding on a toboggan	0.3%	-	4.5%	0.3%	0.3%	-	1.4%	0.5%	0.3%
Turning in place	**78.9%**	0.3%	0.0%	21.4%	-	-	-	0.3%	0.3%
Pedaling	-	**78.5%**	0.0%	-	-	0.3%	.	3.0%	-
Skiing	-	-	**77.4%**	0.3%	0.3%	-	0.3%		0.5%
Turning on a merry-go-round	11.4%	-	-	**71.3%**	0.3%	-	-	0.3%	0.0%
Sitting	-	-	-	-	**70.0%**	-	0.6%	-	44.3%
Playing leapfrog	-	0.9%	-	-	4.8%	**68.8%**	16.6%	0.0%	1.1%
Jumping of a step	-	1.1%	-	-	8.1%	18.8%	**64.9%**	1.1%	1.4%
Climbing	-	3.4%	-	-	-	1.9%	0.3%	**58.6%**	-
Squatting	-	0.0%	-	-	13.9%	-	0.6%	0.0%	**52.2%**
**Total**	**100.0%**	**100.0%**	**100.0%**	**100.0%**	**100.0%**	**100.0%**	**100.0%**	**100.0%**	**100.0%**

Highlighted areas (grey) show incorrect meaning attributed at a level above the chance level (5.5%, i.e. 100/18 choices).

In accordance with our hypothesis, rates of correct recognition were globally high for ASFGs produced by blindfolded sighted. This result suggests a prototypical effect: gesture patterns for each action are both similar enough as a whole yet enough distinct from others to enable participants to arrive at a correct response in a consensual and unambiguous way. Results also showed that recognition rates varied with the type of ASFG. Differences in recognizability were related to differences in the degree of similarity between actions included in the list. As expected, confusions in recognition involved ASFGs which were similar because they are in the same type of action (Turn, Slide or Jump). Logically, well-recognized ASFGs possessed a distinctive attribute that differentiated them from all others: the *Balancing movement* in *Playing on a swing* or the *Back out movement* in *Walking backwards*, for example.

## Experiment 2: Visual recognition of ASFGs produced by late and early blind adults

Experiment 2 investigated the role of visual experience in the production of the types of ASFG and their visual recognition by sighted adults. Thus, its aim was to examine in a different set of 100 sighted adults visual recognition of 18 ASFGs produced by 10 early blind and 7 late blind adults. Experiment 2 would determine if blind participants produced the same patterns of gesture, and if these were as well-recognized as those produced by sighted individuals.

Considering that ASFGs are based on real motor experiences of the legs that are common between the two groups, blind individuals who produced stimuli and sighted individuals who recognized them, our first hypothesis is that ASFGs produced by late and early blind would also obtain good rates of recognition.

However, even if a motor component is expected, the ASFG also possesses a representational purpose (to illustrate an action to someone). According to studies which have shown the rare occurrence of representational gestures in congenitally blind individuals [23–25] we expected that sighted participants would have some difficulty in recognizing ASFGs produced by early blind participants, more so than those produced by late blind participants with previous visual experience.

## Method

### Participants

One hundred sighted subjects (80 women and 20 men, age range = 20–70 years) who had not participated in Experiment 1 responded to new blocks of online surveys presenting videos of gestures produced by late blind and early-blind. Following the same selection procedure used in Experiment 1, our final sample was composed of 91 sighted adult participants (74 women and 17 men, age range 20–70 years). All participants are French speakers, although their nationalities vary (73% french, 20% Swiss and 8% other nationalities). French is the native language for 91% of them. Participants’ responses were collected anonymously.

### Stimuli: Production of 18 ASFGs by blind adults

Ten early blind and 7 late blind adults participated at this stage (see Table 3, Characteristics of blind adults). The group of early blind subjects is composed of 9 women and 1 man, 4 Swiss and 6 French, aged between 28 and 70 (M = 43.5 years, SD = 12.7). Six participants were congenitally blind since birth and 4 became blind before the age of 3. The group of late blind subjects was composed of 1 woman and 6 men, 3 Swiss and 4 French, aged 27 to 69 (M = 47.7, SD = 13.7). Late blind participants became blind at ages ranging from 7 to 37. French participants were recruited with the help of the publishing house “Les Doigts Qui Rêvent” and Swiss participants with the help of “l’Association Suisse pour le Bien des Aveugles”. The study was conducted with the written consent of each participant. This research respects ethical principles for research involving human subjects (World Medical Association Declaration of Helsinki) and was approved by the Swiss Ethic Committee on reseach involving humains. The individuals described in this manuscript have given written informed consent (as outlined in the PLOS consent form) to publish these case details.

**Table 3:** Characteristics of blind adults.

Early blind				
Code	Age of onset of deficit	Age	Cause of deficit	Gender
1	Congenital	50	Unspecified	F
2	Congenital	38	Retinopathy	F
3	Congenital	70	Congenital rubella	F
4	Since the age of 1	43	Optic nerve glioma	F
5	Congenital	37	Leber’s amaurosis	F
6	Congenital	514	Retinopathy	F
7	Since the age of 3	31	Optic nerve glioma	F
8	Congenital	46	Leber’s amaurosis	F
9	Congenital	45	Retinopathy	F
10	Since the age of 2	28	Avitaminosis	M
Late blind				
Code	Age of onset of deficit	Age	Cause of deficit	Gender
1	Since the age of 8	57	Glaucoma	M
2	Since the age of 7	69	Glaucoma	M
3	Since the age of 12	49	Rheumatic disease	M
4	Since the age of 27	38	Glaucoma	M
5	Since the age of 16	41	Unspecified	F
6	Since the age of 10	27	Unspecified	M
7	Since the age of 37	53	Glaucoma	M

The procedure for production of ASFGs by blind adults was identical to that of blindfolded sighted participants. One early blind participant produced no ASFG for *Playing Leapfrog* and *Turning on a merry-go-round*. One late blind produced no ASFG for *Climbing*. They stated they did not know how to simulate these actions with two fingers. Other participants produced all of the 18 action simulations requested. In total, 303 videos (ASFGs made by 17 encoders for 18 actions, minus 3 missing simulations mentioned) were included in the recognition task.

### Experimental condition of the visual recognitiontask

Anticipating that ASFGs produced by blind participants would be less representative than those made by sighted and that the visual recognition task would probably require more decision time, we reduced the number of videos to be viewed in each block in Experiment 2. Contents were divided into 5 blocks; each block contained an equal number of videos per action. Each survey block was viewed by 20 different sighted individuals. Profiles of early and late blind were included in the blocks in a randomized manner.

### Data analysis of visual recognition task

ASFGs with a mean rate of recognition less or more than two standard deviations (2 SD) from the global mean recognition rate by action for their profile group (late blind and early blind) were excluded. As in Experiment 1, arcsin transformation was performed on rates of correct recognition (data in S2 Dataset) and results from both statistical analysis (with ratios data and using arcsin transformation) will be presented.

Preliminary analysis of variance (ANOVAs) was performed to control for gender on rates of recognition of sighted participants in the online survey. Once again, results showed no significant effect on the rates of recognition (Ratio: *F*(1,85) = .03, *p* = .85, η_p_^2^ = <. 001/Arcsin transformation: *F*(1,85) = .01, *p* = .92, η_p_^2^ < .001) and data were further collapsed across these factor.

An 18 (each action) x 5 (response blocks) factorial ANOVA was performed on the mean of action recognition (ratios data and after arcsin transformation) with the actions as within-subjects factors and blocks as between-subjects factors. The block condition was significant (Ratio: *F* (4, 199) = 3.88, *p* < .001, η_p_^2^ = .07/Arcsin transformation: *F* (4, 199) = 3.45, *p* = .005, η_p_^2^ = .07), but the interaction between actions and blocks conditions was not significant (Ratio: *F*(68, 199) = 0.67, *p* = .97, η_p_^2^ = .18/Arcsin transformation = *F*(68, 199) = .85, *p* = .76, η_p_^2^ = .22). Given the fact that the rate of recognition of all actions is the same for all blocks, results were further collapsed across blocks.

## Results and discussion

**Fig 3:**
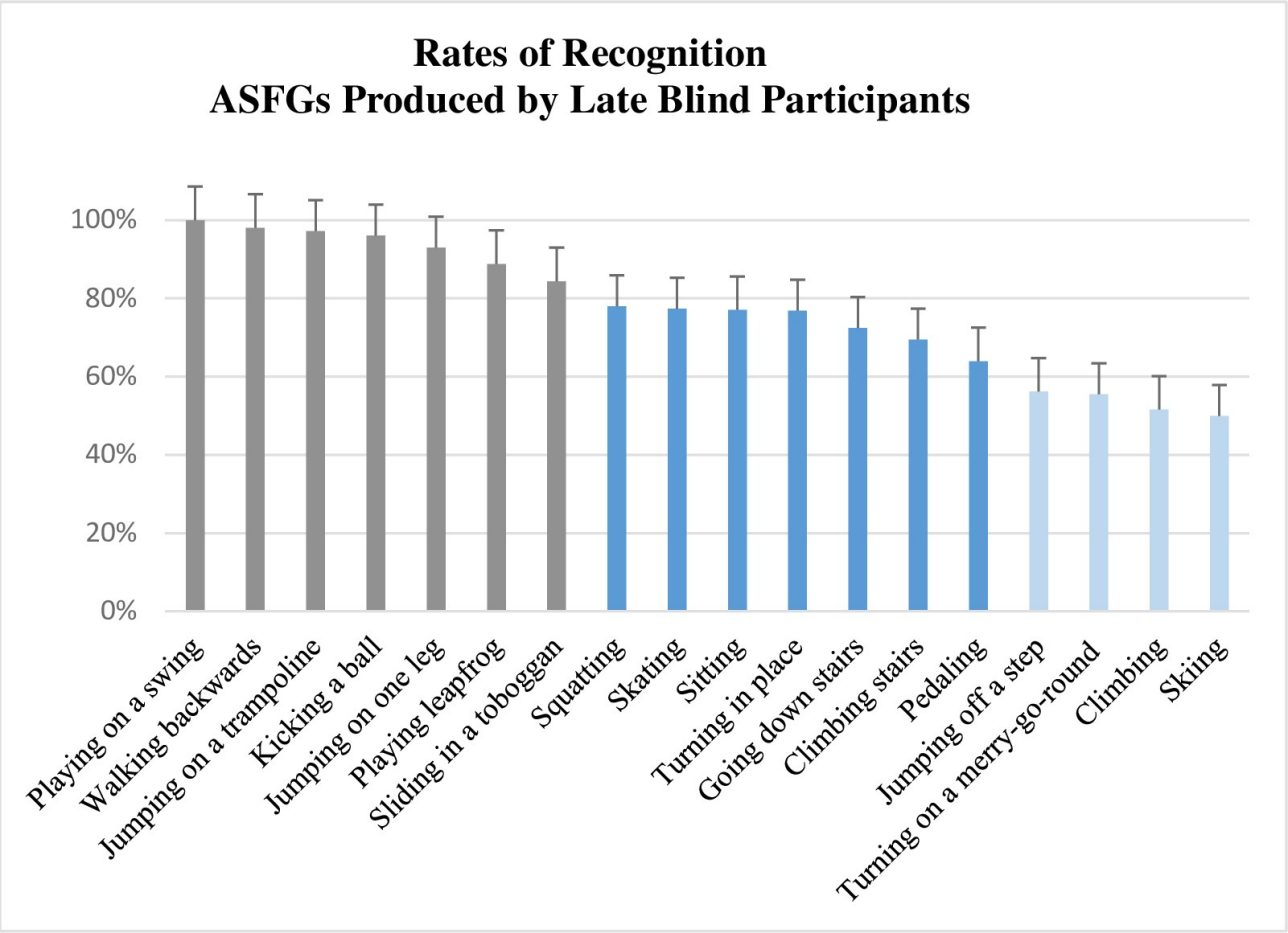
Rates of visual recognition (N = 92) for ASFGs produced by late blind individuals (N = 7). Bar color differenciates rates between 80–100% (grey), between 60–80% (dark blue) and between 60–40% (light blue). The vertical bars represent positive standard deviations. Results with arcsin transformation are available in S1 Appendix.

**Fig 4:**
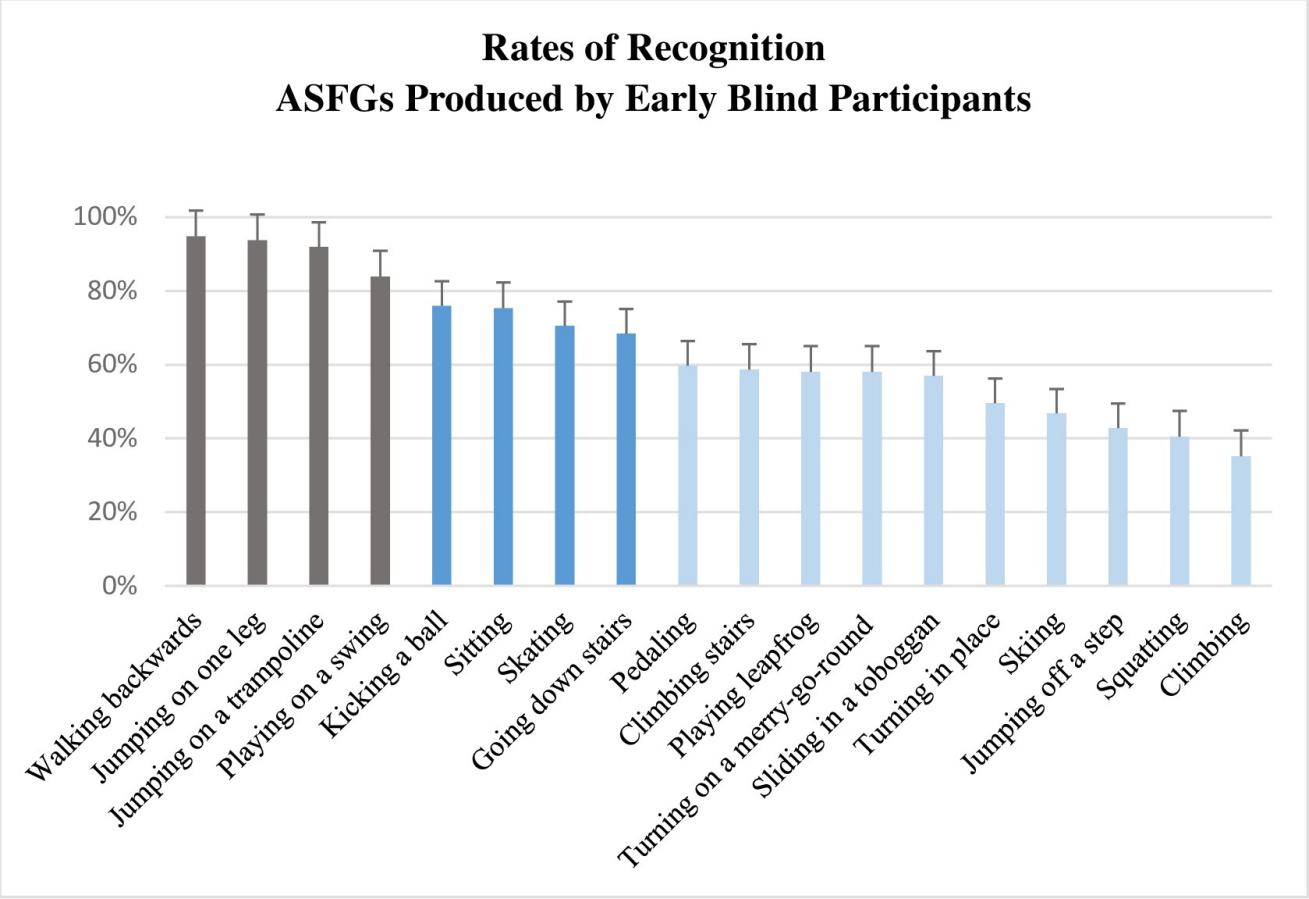
Rates of recognition (N = 92) for ASFGs produced by early blind individuals (N = 10). Bar color differenciates rates between 80–100% (grey), between 60–80% (dark blue) and between 60–40% (light blue). The vertical bars represent positive standard deviations. Results with arcsin transformation are availables in S1 Appendix.

Factorial ANOVA on mean of action recognition by groups showed that the group condition was significant (Ratio: *F*(1, 253) = 24.75, *p* < .001, η_p_^2^ = .09/Arcsin transformation: *F*(1, 253) = 28, *p* < .001, η_p_^2^ = .10). Means of recognition with ratios data and arcsin transformation are availables in S1 Appendix. On average, action simulations produced by late blind individuals were better recognized (M = 77.0%, SD = 16.6%) than those produced by early blind individuals (M = 64.5%, SD = 18.5%). Figs 3 and 4 present the order of recognizability of ASFGs produced by late blind and early blind respectively. Factorial ANOVA also showed that the action condition was significant (Ratio: *F*(17, 253) = 9.58, *p* < .001, η_p_^2^ = .40/Arcsin transformation: *F*(17, 253) = 13, *p* < .001, η_p_^2^ = .46) but interaction between action and group conditions was not significant (Ratio: *F*(17, 253) = 1.24, *p* = .23, η_p_^2^ = .08/Arcsin transformation: *F*(17, 253) = 1.18, *p* = .27, η_p_^2^ = .07). Similar differences in recognizability by action was found in both groups. However, a greater number of ASFGs produced by early blind were situated in the set of recognizability of 60–40% or less.

Tables 4 and 5 present cross-tabulation between the intended meaning and meaning attributed to ASFGs produced by late blind and early blind respectively. Among the 11 actions produced by the group of late blind with a recognition rate below 80% and the 14 actions produced by early blind with a recognition rate below 80%, we observe 18 and 21 occurrences, respectively, of incorrect meaning (in grey) attributed at a level above the level of chance. Observe that certain identity confusions of ASFGs were specific to the group of early blind: *Sliding on a toboggan* was confused with *Skiing* (27%), and vice versa (17%), *Going down stairs* was confused with *Climbing stairs* (13%) and *Jumping off a step* confused with *Going down stairs* (11,3%). Note also that it is only in the case of early blind that an incorrect meaning was attributed more often than the correct intended meaning: *Climbing* is identified as *Climbing* at 36.9% and as *Climbing stairs* at 38.5% and *Squatting i*s identified as *Squatting* at 41% and as Sitting at 49.7%.

**Table 4:** Cross-tabulation of ASFG produced by late blind (columns) and the meaning attributed by participants (rows).

	Squatting	Skating	Sitting	Turning in place	Going down stairs	Climbing stairs	Pedaling	Jumping of a step	Turning on a merry-go-round	Climbing	Skiing
Playing on a swing	-	-	1.6%	-	0.8%	-	3.3%	-	-	1.9%	0.8%
Walking backwards	0.9%	-	0.8%	0.8%	0.8%	-	4.1%	-	0.8%	1.9%	0.8%
Jumping on a trampoline	-	-	-	0.8%	-	0.8%	0.8%	1.6%	-	1.9%	-
Kicking a ball	1.8%	-	0.8%	-	-	-		0.8%	0.8%	-	-
Jumping on one leg	**-**	0.9%	-	3.2%	-	-	0.8%	12.6%	-	-	-
Playing leapfrog	**-**	**-**	-	-	-	-	0.8%	22.8%	-	-	-
Sliding on a toboggan	**-**	**-**	-	-	5.6%	-		2.4%	2.5%	1.9%	9.4%
Squatting	**77.3%**	**-**	14.7%	-	-	-	0.8%	-	-		
Skating	-	**77.8%**	0.0%	11.2%	3.2%	-	8.1%	-	17.4%	0.9%	38.6%
Sitting	20.0%	-	**76.7%**	0.0%	0.8%	-		-			-
Turning in place	-	-	-	**77.6%**	-	-	0.8%	-	19.0%		-
Going down stairs	-	1.9%	-	-	**74.4%**	-	6.5%	0.8%	-	0.9%	0.8%
Climbing stairs	-	-	-	-	2.4%	**70.3%**	6.5%	-	-	33.0%	-
Pedaling	-	0.9%	-	-	3.2%	0.8%	**64.2%**	0.8%	1.7%	4.7%	-
Jumping of a step	-	-	0.8%	-	2.4%	0.8%		**57.5%**	-	0.9%	-
Turning on a merry-go-round	-	-	-	6.4%	-	-	0.8%	-	**57.0%**	-	-
Climbing	-	-	2.3%	-	-	26.6%	1.6%	-	-	**50.9%**	-
Skiing	-	18.5%	2.3%	-	6.4%	0.8%	0.8%	0.8%	0.8%	0.9%	**49.6%**
**Total**	**100.0%**	**100.0%**	**100.0%**	**100.0%**	**100.0%**	**100.0%**	**100.0%**	**100.0%**	**100.0%**	**100.0%**	**100.0%**

Highlight areas (grey) shows incorrect meaning attributed above the chance level (5.5%, i.e. 100/18 choices).

**Table 5:** Cross-tabulation of ASFG produced by early blind (columns) and the meaning attribuated by participants (rows).

	Kicking a ball	Sitting	Skating	Going down stairs	Pedaling	Climbing stairs	Playing leapfrog	Turning on a merry-go-round	Sliding on a toboggan	Turning in place	Skiing	Jumping of a step	Squatting	Climbing
Walking backwards	-	1.9%	-	3.3%	1.8%	-	-	-	-	1.1%	0.0%	0.6%	0.6%	-
Jumping on one leg	4.2%	-	-	1.6%	1.8%	2.2%	3.6%	-	3.3%	0.5%	0.5%	8.5%	0.6%	5.0%
Jumping on a trampoline	9.1%	0.6%	-	-	0.6%	3.8%	6.6%	-	1.6%	0.5%	-	3.4%	-	2.2%
Playing on a swing	3.0%	0.6%	-	0.5%	2.4%	0.5%	-	1.8%	1.6%	-	0.5%	-	0.6%	-
Kicking a ball	**76.4%**	1.3%	0.6%	-	3.0%	-	0.6%	4.2%	0.5%	1.6%	-	-	2.2%	1.1%
Sitting	-	**75.5%**	-	-	-	-	1.2%	-	-	0.5%	0.5%	-	49.7%	-
Skating	2.4%	1.9%	**70.8%**	1.1%	3.0%	1.6%	-	9.0%	2.2%	10.3%	34.1%	-	-	2.8%
Going down stairs	-	-	1.2%	**67.6%**	1.2%	2.7%	-	-	-	1.1%	1.1%	11.3%	-	2.8%
Pedaling	-	0.6%	2.4%	3.3%	**59.4%**	2.2%	0.6%	-	-	-	-	3.4%	-	1.1%
Climbing stairs	-	-	3.6%	13.2%	4.8%	**59.2%**	1.2%	-	1.1%	-	-	2.3%	-	38.5%
Playing leapfrog	1.2%	0.6%	-	1.6%	1.8%	1.1%	**57.8%**	-	1.6%	1.6%	-	26.6%	1.1%	1.1%
Turning on a merry-go-round	0.6%	-	-	-	4.2%	-	0.0%	**59.9%**	0.0%	30.4%	-	-	-	-
Sliding on a toboggan	1.2%	1.3%	2.4%	0.5%	0.6%	-	1.2%	-	**54.9%**	0.5%	17.0%	0.6%	-	2.8%
Turning in place	-	0.6%	0.6%	-	6.1%	-	-	22.8%		**49.5%**	**0.5%**	-	-	-
Skiing	1.2%	-	17.9%	0.5%	0.6%	-	-	2.4%	27.5%	1.6%	**45.1%**	1.1%	-	-
Jumping of a step	0.6%	-		3.3%	-	0.5%	24.1%	-	2.7%	-	0.5%	**42.4%**	2.2%	5.6%
Squatting	-	15.1%		0.0%	-	-	0.6%	-	-	-	-	-	41.4%	-
Climbing	-	-	0.6%	3.3%	8.5%	26.1%	2.4%	-	2.7%	0.5%	-	-	1.7%	**36.9%**
**Total**	**100.0%**	**100.0%**	**100.0%**	**100.0%**	**100.0%**	**100.0%**	**100.0%**	**100.0%**	**100.0%**	**100.0%**	**100.0%**	**100.0%**	**100.0%**	**100.0%**

Highlight areas (grey) shows incorrect meaning attributed above the chance level (5.5%, i.e. 100/18 choices).

## Results and discussion

The results observed in Experiments 1 and 2 are globally similar: a very high level of visual recognition and a very similar order of recognizability among the 18 actions. In fact, the recognition rates obtained by ASFGs produced by early blind individuals are quite good (64.5%) though less accurate than those of late blind individuals, which are excellent (77.0%) and very close to results obtained by ASFGs produced by sighted individuals (81.5%). Results from cross-tabulations between the intended meaning of the ASFG and the meaning attributed by participants in the recognition task revealed that some incorrect meanings were specific to the ASFG produced by the early blind group.

## Experiment 3: Additional evaluation by two independent judges of prototypical and atypical attributes of ASFGs produced by blindfolded sighted, late blind and early blind individuals

In order to match data of production with data of recognition obtained in Experiment 3 two independent judges were asked to evaluate and classify each ASFG as prototypical or atypical in regard to their similarity with a prototypical exemplar. This procedure of codage is based on Rosh and Mervis's theory of family resemblances [27]. In sum, members of a category come to be viewed as typical exemples in proportion to how many attributes they have in common with other members. A prototypical member is one who has the most attributes in common with other members of the category and the fewest attributes in common with other categories. In a connected field, a study about the recognition and production of tactile geometric shapes showed that prototypical effects depend on visual experience, given that no occurrence of this effect was found in congenitally blind adults in a tactile task [28]. In Experiment 3, we expected the ASFGs produced by three groups (and more particularly the ASFGs produced by blindfolded sighted individuals) would be evaluated as globally protypical. Following results obtained in Experiments 1 and 2, we also expected that ASFGs judged as more protoypical would be those that possesses a distinctive attribute that differentiated them from all others (e.g. *Playing on a swing* or *Walking backwards)*. However, some evaluations of atypical attributes of ASFG produced by blind individuals can be expected among specific actions in which ambiguous meaning was attributed in Experiment 2 (e.g. *Sliding on a toboggan* or *Going down stairs*). To test these hypotheses, each judge was asked to evaluate each of 663 videos of ASFGs produced by three groups. For ASFGs judged as atypical, judges were asked to describe attributes that cause problems.

## Method

### Participants

Two research assistants, Swiss University of Geneva Master's students in Psychology aged 23 and 25, were recruited to evaluate all ASFGs produced by blindfolded sighted, late blind and early blind individuals in Experiments 1 and 2. They were unaware of the purpose of the experiment and the experimental conditions.

### Experimental condition

In total, each judge looked at 663 actions (e.g. 37 ASFG x18 actions = 666 minus 3 missing ASFGs in the group of blind individuals as indicated in Experiment 2). First, they were instructed to look at all the videos of ASFGs for each action set: 37 ASFGs in total by action set with 20 produced by sighted, 10 by early blind and 7 by late blind participants in each action set). They were not preliminary informed about the visual status of ASFG encoders. Then, they were asked to classify each ASFG as typical or atypical in regard to their similarity with a prototypical exemplar. For ASFGs judged as atypical, judges were asked to write attributes that cause problems (data in S3 Dataset). Global percentage agreement between the two judges was 79%.

## Results and discussion

Table 6 presents prototypical and atypical ASFGs produced by blindfolded sighted, early blind and late blind individuals. Highlighted areas (grey) present a description of protopypical ASFG and how many times ASFGs produced by each group (columns) were considered by judges as prototypical. White lines presented how many times judges considered ASFG as atypical with examples of atypical attributes listed.

**Table 6:** Prototypical and atypical ASFGs produced by blindfolded sighted, early blind and late blind individuals.

**ASFG**	** **	**Blindfolded Sighted**	**Early Blind**	**Late Blind**
**Playing on a swing**	**Prototypical ASFG**	**Index and middle fingers swing in the air**		
		**13/20**	**0/10**	**4/7**
	**Nb of atypical ASFG**	0/20	6/10	1/7
	**Example of atypical attributes**		Vertical jump / Swing with whole arms	Vertical jump
**ASFG**	** **	**Blindfolded Sighted**	**Early Blind**	**Late Blind**
**Kicking a ball**	**Prototypical ASFG**	**Kick with index and middle fingers remaining on the table**		
** **		**16/20**	**2/10**	**3/7**
** **	**Atypical ASFG**	1/20	5/10	2/7
** **	**Example**	Kick with two fingers at the same time	Kick with hands/ use of other hand as a ball	Kick with two fingers at the same time
**ASFG**		**Blindfolded Sighted**	**Early Blind**	**Late Blind**
**Pedaling**	**Prototypical ASFG**	**Intercalated circular movement of index and middle fingers**		
		**16/20**	**2/10**	**3/7**
	**Atypical ASFG**	1/20	2/10	2/7
	**Example**	Irregular movement	Pedaling with one finger on each hand	Irregular movement
**ASFG**	** **	**Blindfolded Sighted**	**Early Blind**	**Late Blind**
**Climbing**	**Prototypical ASFG**	**Index and middle climb movement**		
		**10/20**	**2/10**	**0/7**
	**Atypical ASFG**	6/20	6/10	3/7
	**Example**	Irregular climb	Fingers remain on the table/ Use of other hand as a climbing wall	Irregular climb
		**Blindfolded Sighted**	**Early Blind**	**Late Blind**
**Sliding on a toboggan**	**Prototypical ASFG**	**Slide movement of index and middle fingers from top to bottom**		
		**12/20**	**3/10**	**5/7**
	**Atypical ASFG**	5/20	6/10	1/7
	**Example**	Slide movement that goes back up	Fingers remain on the table	Use of other hand as a "toboggan support"
		**Blindfolded Sighted**	**Early Blind**	**Late Blind**
**Jumping on a trampoline**	**Prototypical ASFG**	**Vertical straight jump with index and middle fingers**		
		**18/20**	**7/10**	**3/7**
	**Atypical ASFG**	1/20	2/10	1/7
	**Example**	pirouettes on the air	Jump with whole hand	Use of other hand as a trampoline support
		**Blindfolded Sighted**	**Early Blind**	**Late Blind**
**Playing leapfrog**	**Prototypical ASFG**	**Jump with two wide index and middle fingers while moving**		
		**11/20**	**2/10**	**3/7**
	**Atypical ASFG**	4/20	5/10	3/7
	**Example**	Use of the other hand as a leapfrog barrier	Use of the other hand as a leapfrog barrier	Use of the other hand as a leapfrog barrier
		**Blindfolded Sighted**	**Early Blind**	**Late Blind**
**Jumping on one leg**	**Prototypical ASFG**	**Jump with only the index finger while moving**		
		**17/20**	**8/10**	**7/7**
	**Atypical ASFG**	0/20	1/10	0/7
	**Example**		Jump with two fingers	
** **	** **	**Blindfolded Sighted**	**Early Blind**	**Late Blind**
**Jumping off a step**	**Prototypical ASFG**	**Only one jump with index and middle fingers**		
		**9/20**	**1/10**	**2/10**
	**Atypical ASFG**	2/20	5/10	3/7
	**Example**	Use of other hand as a step	Use of the edge of the table or the other hand as a step	Use of the edge of the table or the other hand as a step
** **	** **	**Blindfolded Sighted**	**Early Blind**	**Late Blind**
**Squatting**	**Prototypical ASFG**	**Distal phalanges put on the table**		
		**15/20**	**4/10**	**6/10**
	**Nb of atypical ASFG**	1/20	4/10	1/7
	**Example**	Gesture in air	Two hand gestures	Stiff fingers
		**Blindfolded Sighted**	**Early Blind**	**Late Blind**
**Sitting**	**Prototypical ASFG**	**Inclination of proximal phalanges**		
		**12/20**	**4/10**	**3/7**
	**Atypical ASFG**	4/20	3/10	2/7
	**Example**	Use of the other hand as a seat/ Gesture on the air	Use of the other hand as a seat/ stiff fingers	Use of the other hand as a seat
		**Blindfolded Sighted**	**Early Blind**	**Late Blind**
**Turning on a merry-go-round**	**Prototypical ASFGs**	**Index and middle fingers slide in a circular path**		
		**10/20**	**3/10**	**1/7**
	**Atypical ASFGs**	3/20	5/10	3/7
	**Example**	Hand turns in place	Hand turns in place/hand does not turn	Hand does not turn
		**Blindfolded Sighted**	**Early Blind**	**Late Blind**
**Turning in place**	**Prototypical ASFGs**	**Vertical fingers and whole hand turn in place**		
		**10/20**	**3/10**	**1/7**
	**Atypical ASFGs**	6/20	3/10	4/7
	**Example**	Hand does not turn	Hand does not turn/use of other hand as a referential cue to the circular path	Hand does not turn
		**Blindfolded Sighted**	**Early Blind**	**Late Blind**
**Skating**	**Prototypical ASFGs**	**Index and middle fingers slide one by one**		
		**13/20**	**5/10**	**2/7**
	**Atypical ASFGs**	3/20	2/10	3/7
	**Example**	Walk rather than slide	Slide with one finger on each hand	Walk rather than slide
		**Blindfolded Sighted**	**Early Blind**	**Late Blind**
**Skiing**	**Prototypical ASFGs**	**Index and middle fingers slide in a serpentine path**		
		**11/20**	**5/10**	**2/7**
	**Atypical ASFGs**	5/20	3/10	0/7
	**Example**	Little jump after each slide movement	Slide with one finger on each hand	
		**Blindfolded Sighted**	**Early Blind**	**Late Blind**
**Going down stairs**	**Prototypical ASFG**	**Index and middle fingers going down one by one**		
		**14/20**	**6/10**	**3/7**
	**Atypical ASFGs**	5/20	3/10	4/7
	**Example**	Use of the other hand as a stair	Fingers remain on the table	Fingers remain on the table /use of other hand as a stair
		**Blindfolded Sighted**	**Early Blind**	**Late Blind**
**Climbing stairs**	**Prototypical ASFG**	**Index and middle fingers climbing one by one**		
		**16/20**	**6/10**	**3/7**
	**Atypical ASFGs**	2/20	4/10	3/7
	**Example**	Use of the other hand as a stair	Fingers remain on the table	Fingers remain on the table/Use of other hand as a stair
		**Blindfolded Sighted**	**Early Blind**	**Late Blind**
**Walking backwards**	**Prototypical ASFGs**	**Index and middle fingers going backwards one by one**		
		**19/20**	**6/10**	**5/7**
	**Atypical ASFG**	0/20	2/10	1/7
	**Example**		Irregular walk	Irregular walk
		**Blindfolded Sighted**	**Early Blind**	**Late Blind**
**Total**	**Prototypical ASFG**	**243/360**	**70/178**	**55/125**
	Atypical ASFG	50/360	67/178	37/125

As expected, the first result, the 18 ASFGs that obtained a high level of recognition in Experiments 1 and 2 by action, has been judged as a prototype *(Jumping on one leg*, *Playing on a swing*, *Walking backward)*. 68%, 39% and 44% ASFGs produced by blindfolded sighted, early blind and late blind individuals, respectively were coded as prototypical by judges and 14%, 37% and 29% were coded as atypical. The remainer of the ASFGs produced did not obtain an agreement from judges (e.g. 18% for blindfolded sighted, 23% for early blind and 26% for late blind). Overall, these results indicated that ASFGs produced by blindfolded sighted individuals were more prototypical than those produced by early and late blind individuals. Here the prototypical affect is influenced by the visual status of ASFG producers.

It is interesting to observe that in actions such as *Playing on a swing*, *Pedaling and Skating*, only the early blind group produced the gesture with two hands or two arms. We argue that here, they produce a gesture rather from a *character-viewpoint* than from an *observer- viewpoint*. Concerning the simulation of the action *Sliding on a toboggan*, even if early blind, late blind and sighted individuals produced the same finger gesture pattern (i.e. fingers that slide), 6 to 10 of early blind did not raise their hand. Their two fingers remained on a surface simulating only the motor experience of the body which slides in contact with the toboggan's surface. It is important to note that this simulation is perfectly relevant to a tactilo-kinesthetic experience. When we slide on a toboggan, our body stays in contact with the toboggan throughout the entire slide. The same behavior was identified in the actions *Going down stairs* and *Climbing Stairs*, also presented in ASFG produced by late blind individuals in this case. Some atypical attributes of ASFG produced by early blind and, to lesser extent, late blind individuals seem to be more centred in the tactile-kinesthesic or haptic experience of the body.

## General discussion

**Fig 5:**
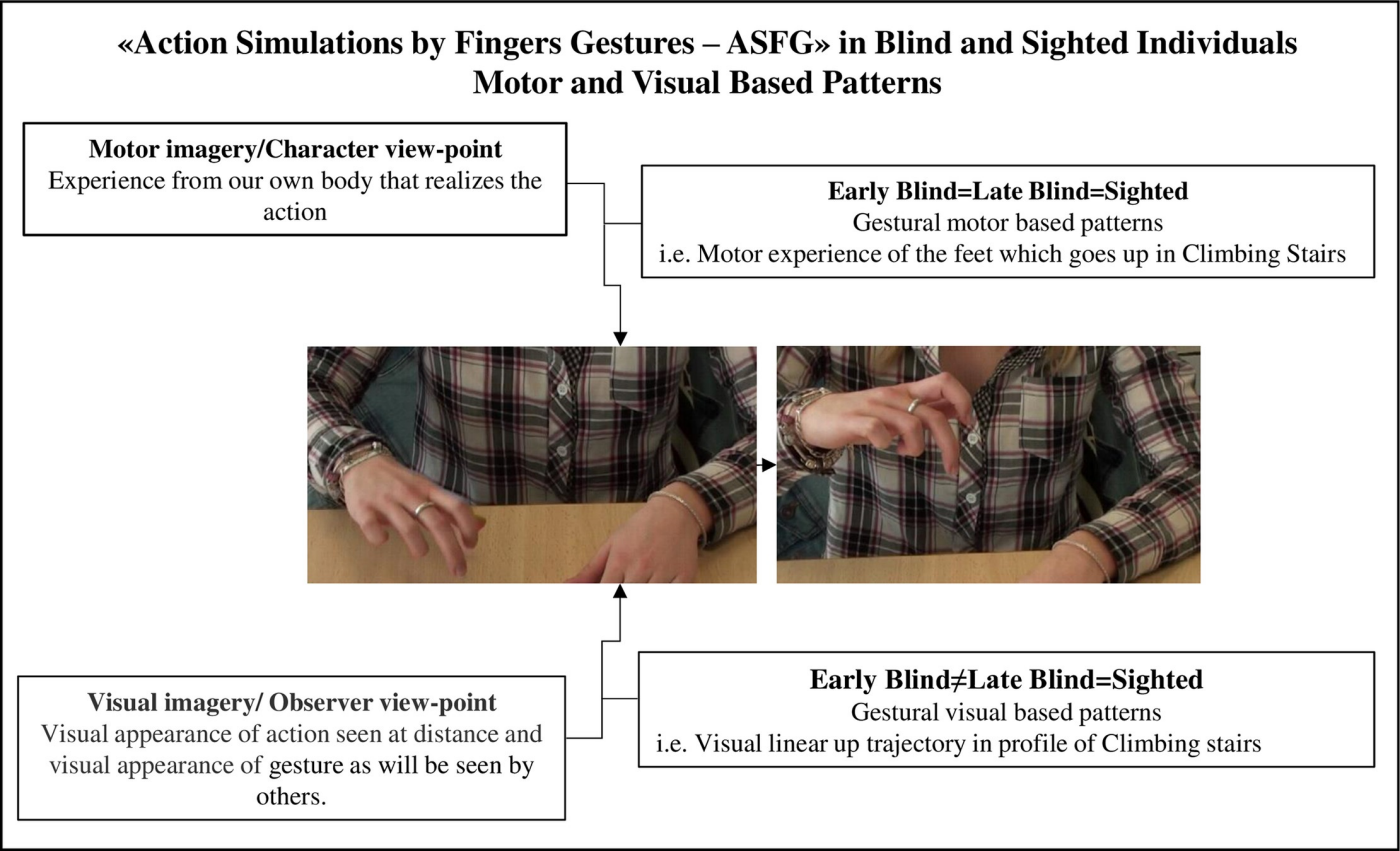
Action simulations by finger gestures in blind and sighted individuals: Motor and visually based patterns.

Despite some atypical attributes found to a greater degree in ASFGs produced by early blind individuals, all three groups obtained good rates of recognition in Experiments 1 and 2. In Experiment 3, the percentage of ASFGs coded as atypical by both judges is 68% for blindfolded sighted producers, 39% for early blind producers and 44% for late blind producers. Qualitative and descriptive analysis of atypical attributes showed that ASFGs produced by early blind individuals convey more a *character-viewpoint* and less the visual norms of gestural communication. These results could explain why ASFGs produced by early blind participants were less recognized by sighted adults in Experiment 2 and also why they were more coded as atypical by two independent judges in Experiment 3. The results obtained corroborate those of other studies showing that representational components are rare in the gestural activity of individuals who have never seen or became blind quite early [23–25]. This representational component resulting from *observer-viewpoint*, concerns the visual appearance of the action seen at a distance and/or the visual appearance of the gestures (as will be seen by others). These gestural visual patterns are influenced by visual experience. This explains why sighted participants sometimes have more difficulty recognizing ASFGs produced by blind participants, even though gestural motor based patterns are very similar between groups. This idea is schematized in Fig 5.

Due to the nature of the gestures studied (simulation of the legs with fingers), a substantial part of *character-viewpoint* gestures [5] resulting from motor based patterns was expected. High rates of recognition for ASFGs produced by all three groups suggested that gestural motor based patterns of ASFGs are very similar among early blind, late blind and sighted individuals (i.e., motor experience of the feet which go up in *Climbing Stairs*). Differences in recognition rates obtained in Experiments 1 and 2 seems to be related to the visual and representational component of gesture.

To conclude, the results of Experiments 1 and 2 may have important practical implications. Despite some differences related to the visual rules of gesturing, the ASFGs examined seem to be a meaningful simulation procedure for both blind and sighted individuals. The next step of the research project is to design 3D illustrations engaging the common ASFGs observed. More recognized ASFGs produced by all three groups will be selected and prototypes will test the haptic recognition of these devices by blind and sighted children [29]. A feasibility study is already underway with our editorial partnership (Les Doigts Qui Rêvent, www.ldqr.org) to evaluate the possibilities of including ASFGs in tactile devices such as children's books but also in mobility aids. We are also currently exploring the field of new technologies to develop and test new multimodal interfaces to associate ASFGs with sound feedback in 3D scenarios. The advantage of 3D scenarios that engage ASFGs over traditional 3D representations (toys or miniature objects already commonly used in this field of intervention) is the reactivation of motor components from real interactions with objects (enactment effect). These 3D illustrations involving ASFGs seem to open a promising design path in the field of visual disability. Exploring new relationships between representational devices and common sensorimotor experiences between individuals helps develop the design of new communication interfaces that are straightfowardly understood by a larger number of subjects, regardless of their sensory abilities.

## Supporting information

S1 Dataset Recognition means experiment 1. (XLSX)

S2 Dataset Recognition means experiment 2. (XLSX)

S3 Dataset Codage by two independent judges experiment 3. (XLSX)

S1 Appendix Global results. (DOCX)
