# Supplementary material for: Adults’ visual recognition of actions simulations by finger gestures (ASFGs) produced by sighted and blind individuals
Source: PLoS One. 2019 Mar 28;14(3):e0214371. doi: 10.1371/journal.pone.0214371 (PMC6438591; doi:10.1371/journal.pone.0214371)
Supplement: S1 Appendix — (DOCX) [file pone.0214371.s004.docx]

**S1 Appendix**

**Global results: rates of recognition (%) and rates after arcsin transformation**

**Experiment 1**

- **Table 1. Rates of visual recognition for ASFG produced by blindfolded sighted individuals**

**Experiment 2**

- **Table 2. Rates of visual recognition for ASFG produced by late blind individuals**
- **Table 3. Rates of visual recognition for ASFG produced by early blind individuals**

| **Table 1. Rates of visual recognition for ASFG produced by blindfolded sighted individuals** | | | | | |
| --- | --- | --- | --- | --- | --- |
|  |  | **Rates of recognition (%)** | | **Arcsin tranformation** | |
| **Item** | **Action** | **M** | **SD** | **M2** | **SD** |
| 1 | Playing on a swing | 99.2% | 3.7% | 1.54159575 | 0.06345784 |
| 2 | Kicking a ball | 93.3% | 3.7% | 1.27655223 | 0.06345784 |
| 3 | Pedaling | 79.1% | 3.7% | 1.00451678 | 0.06345784 |
| 4 | Climbing | 59.9% | 3.6% | 0.68641126 | 0.06185106 |
| 5 | Sliding on a toboggan | 80.3% | 3.8% | 0.98351555 | 0.06519674 |
| 6 | Jumping on a trampoline | 92.8% | 3.6% | 1.29472911 | 0.06185106 |
| 7 | Playing leapfrog | 68.8% | 3.6% | 0.82036528 | 0.06185106 |
| 8 | Jumping on one leg | 96.0% | 3.7% | 1.39281327 | 0.06345784 |
| 9 | Jumping off a step | 65.2% | 3.7% | 0.72190104 | 0.06345784 |
| 10 | Squatting | 52.8% | 3.6% | 0.61255336 | 0.06185106 |
| 11 | Sitting | 69.8% | 3.9% | 0.79223602 | 0.06708689 |
| 12 | Turning on a merry-go-round | 72.5% | 3.7% | 0.86109607 | 0.06345784 |
| 13 | Turning in place | 79.3% | 3.7% | 0.96129624 | 0.06345784 |
| 14 | Skating | 87.2% | 3.8% | 1.08845844 | 0.06519674 |
| 15 | Skiing | 77.8% | 3.8% | 0.97113771 | 0.06519674 |
| 16 | Going down stairs | 95.0% | 3.9% | 1.34597309 | 0.06708689 |
| 17 | Climbing stairs | 82.0% | 3.7% | 1.01503606 | 0.06345784 |
| 18 | Walking backwards | 99.0% | 3.6% | 1.50603586 | 0.06185106 |
| **Total** | | **80.5%** | **13.8%** | **1.04867906** | **0.28442773** |

| **Table 2. Rates of visual recognition for ASFG produced by late blind individuals** | | | | | |
| --- | --- | --- | --- | --- | --- |
|  |  | **Rates of recognition (%)** | | **Arcsin tranformation** | |
| **Item** | **Action** | **M** | **SD** | **M2** | **SD** |
| 1 | Playing on a swing | 100.0% | 8.5% | 1.57079633 | 0.12870763 |
| 2 | Kicking a ball | 96.0% | 7.9% | 1.37440597 | 0.12870763 |
| 3 | Pedaling | 64.0% | 8.5% | 0.72368938 | 0.11916011 |
| 4 | Climbing | 51.6% | 8.5% | 0.55479185 | 0.12870763 |
| 5 | Sliding on a toboggan | 84.4% | 8.5% | 1.0281462 | 0.11916011 |
| 6 | Jumping on a trampoline | 97.2% | 7.9% | 1.4029692 | 0.12870763 |
| 7 | Playing leapfrog | 88.8% | 8.5% | 1.10409988 | 0.12870763 |
| 8 | Jumping on one leg | 92.9% | 7.9% | 1.28976606 | 0.11916011 |
| 9 | Jumping off a step | 56.2% | 8.5% | 0.64821488 | 0.11916011 |
| 10 | Squatting | 77.9% | 7.9% | 0.90468434 | 0.12870763 |
| 11 | Sitting | 77.1% | 8.5% | 0.94148429 | 0.11916011 |
| 12 | Turning on a merry-go-round | 55.5% | 7.9% | 0.62792537 | 0.11916011 |
| 13 | Turning in place | 76.9% | 7.9% | 0.89171859 | 0.11916011 |
| 14 | Skating | 77.4% | 7.9% | 0.90350866 | 0.12870763 |
| 15 | Skiing | 50.0% | 7.9% | 0.57251096 | 0.11916011 |
| 16 | Going down stairs | 72.4% | 7.9% | 0.99431184 | 0.11916011 |
| 17 | Climbing stairs | 69.5% | 7.9% | 0.80695387 | 0.11916011 |
| 18 | Walking backwards | 98.1% | 8.5% | 1.45666825 | 0.12870763 |
| **Total** | | **77.0%** | **16.6%** | **0.98870255** | **0.00488174** |

| **Table 3. Rates of visual recognition for ASFG produced by early blind individuals** | | | | | |
| --- | --- | --- | --- | --- | --- |
|  |  | **Rates of recognition (%)** | | **Arcsin tranformation** | |
| **Item** | **Action** | **M** | **SD** | **M2** | **SD** |
| 1 | Playing on a swing | 83.8% | 7.0% | 1.05435408 | 0.10508934 |
| 2 | Kicking a ball | 76.0% | 6.6% | 0.94579839 | 0.10508934 |
| 3 | Pedaling | 59.8% | 6.6% | 0.65957005 | 0.10508934 |
| 4 | Climbing | 35.2% | 7.0% | 0.38357723 | 0.0996965 |
| 5 | Sliding on a toboggan | 57.0% | 6.6% | 0.63727448 | 0.0996965 |
| 6 | Jumping on a trampoline | 91.9% | 6.6% | 1.22394022 | 0.10508934 |
| 7 | Playing leapfrog | 58.1% | 7.0% | 0.70902221 | 0.10508934 |
| 8 | Jumping on one leg | 93.7% | 7.0% | 1.31172639 | 0.10508934 |
| 9 | Jumping off a step | 42.8% | 6.6% | 0.47490982 | 0.0996965 |
| 10 | Squatting | 40.5% | 7.0% | 0.43230773 | 0.0996965 |
| 11 | Sitting | 75.3% | 7.0% | 0.91094128 | 0.10508934 |
| 12 | Turning on a merry-go-round | 58.1% | 7.0% | 0.65808507 | 0.10508934 |
| 13 | Turning in place | 49.6% | 6.6% | 0.54240551 | 0.0996965 |
| 14 | Skating | 70.5% | 6.6% | 0.81347765 | 0.10508934 |
| 15 | Skiing | 46.8% | 6.6% | 0.51567773 | 0.0996965 |
| 16 | Going down stairs | 68.4% | 6.6% | 0.88795077 | 0.0996965 |
| 17 | Climbing stairs | 58.6% | 7.0% | 0.67614566 | 0.0996965 |
| 18 | Walking backwards | 94.8% | 7.0% | 1.32225811 | 0.0996965 |
| **Total** | | **64.5%** | **18.5%** | **0.78663458** | **0.10239292** |
